# Fine Mapping of Virescent Leaf Gene *v-1* in Cucumber (*Cucumis sativus* L.)

**DOI:** 10.3390/ijms17101602

**Published:** 2016-09-22

**Authors:** Han Miao, Shengping Zhang, Min Wang, Ye Wang, Yiqun Weng, Xingfang Gu

**Affiliations:** 1 Institute of Vegetables and Flowers, Chinese Academy of Agricultural Sciences, Beijing 100081, China; miaohan@caas.cn (H.M.); zhangshengping@caas.cn (S.Z.); w.jsun@163.com (M.W.); wangye@caas.cn (Y.W.); 2 USDA-ARS Vegetable Crops Research Unit, Horticulture Department, University of Wisconsin, Madison, WI 53706, USA

**Keywords:** cucumber, virescent leaf, chloroplast development, map-based cloning

## Abstract

Leaf color mutants are common in higher plants that can be used as markers in crop breeding or as an important tool in understanding regulatory mechanisms in chlorophyll biosynthesis and chloroplast development. In virescent leaf mutants, young leaves are yellow in color, which gradually return to normal green when the seedlings grow large. In the present study, we conducted phenotypic characterization and genetic mapping of the cucumber virescent leaf mutant 9110Gt conferred by the *v-1* locus. Total chlorophyll and carotenoid content in 9110Gt was reduced by 44% and 21%, respectively, as compared with its wild type parental line 9110G. Electron microscopic investigation revealed fewer chloroplasts per cell and thylakoids per chloroplast in 9110Gt than in 9110G. Fine genetic mapping allowed for the assignment of the *v-1* locus to a 50.4 kb genomic DNA region in chromosome 6 with two flanking markers that were 0.14 and 0.16 cM away from *v-1*, respectively. Multiple lines of evidence supported *CsaCNGCs* as the only candidate gene for the *v-1* locus, which encoded a cyclic-nucleotide-gated ion channel protein. A single nucleotide change in the promoter region of *v-1* seemed to be associated with the virescent color change in 9110Gt. Real-time PCR revealed significantly lower expression of *CsaCNGCs* in the true leaves of 9110Gt than in 9110G. This was the first report that connected the *CsaCNGCs* gene to virescent leaf color change, which provided a useful tool to establish linkages among virescent leaf color change, chloroplast development, chlorophyll biosynthesis, and the functions of the *CsaCNGCs* gene.

## 1. Introduction

In higher plants, there are diverse leaf color mutations such as albino, xanthan, light green, virescent, stripes, zebra, and stay-green [1,2]. Due to the ease of identification, large numbers of leaf-color mutants have been described and characterized in many plant species like *Arabidopsis thaliana* [3], barley [4], maize [5], rice [6,7,8], soybean [9], sunflower [10], and wheat [11,12]. For example, in rice, more than 200 leaf color mutants have been documented, and 154 responsible genes have been mapped to all 12 chromosomes, of which 53 have been cloned [13]. In soybean, 25 nuclear genes affecting chlorophyll-deficiency have been identified and mapped [9]. Leaf-color mutants have also been reported in tomato [14], pepper [15], and carrots [16,17]. Investigation of these color mutants has contributed significantly to our understanding of chlorophyll metabolism and chloroplast development [18]. On the practical side, these mutations are also useful in plant breeding such as identification of genetic purity in hybrid production [19].

Virescent color is a special color mutation. Plants with this kind of mutation exhibit a delay in greening up to the first true leaf due to a delay in chloroplast development; which is indistinguishable from the wild type (WT) when mature. The first recessive virescent leaf mutant was described in maize in 1912 [20]. Virescent mutants have been characterized in barley (*Hordeum vulgare* L.) [21], common bean (*Phaseolus vulgaris*) [22], cotton [23], peanut [24], maize [25], Arabidopsis [20], and rice [26]. The Vir-C mutant of tobacco also displays a virescent phenotype, but it is maternally inherited [27].

In cucumber (*Cucumis sativus* L.), several leaf color mutants have been reported including chlorophyll deficient (*cd*), golden leaves (*g*), golden cotyledon (*gc*), light green cotyledons-1 (*lg-1*), light green cotyledons-2 (*lg-2*), virescent (*v*), variegated virescent (*vvi*), yellow cotyledons-1 (*yc-1*), yellow cotyledons-2 (*yc-2*), yellow plant (*yp*), yellow stem (*ys*) [28], and virescent (*v-1*) [29], The *yp* locus has recently been cloned which encodes a magnesium chelatase submit unit I [30]. The cucumber virescent leaf mutant 9110Gt has been phenotypically characterized. Genetically, this mutant was shown to be controlled by a single recessive gene *v-1* which was mapped to cucumber chromosome 6 [31]. The objective of the present study was to clone the candidate gene for the *v-1* mutant. We carried out high-resolution genetic mapping and identified the most likely candidate gene for the *v-1* locus using the cucumber genome and molecular marker resources.

## 2. Results

### 2.1. Phenotypic Characterization of the v-1 Mutant

**Figure 1:**
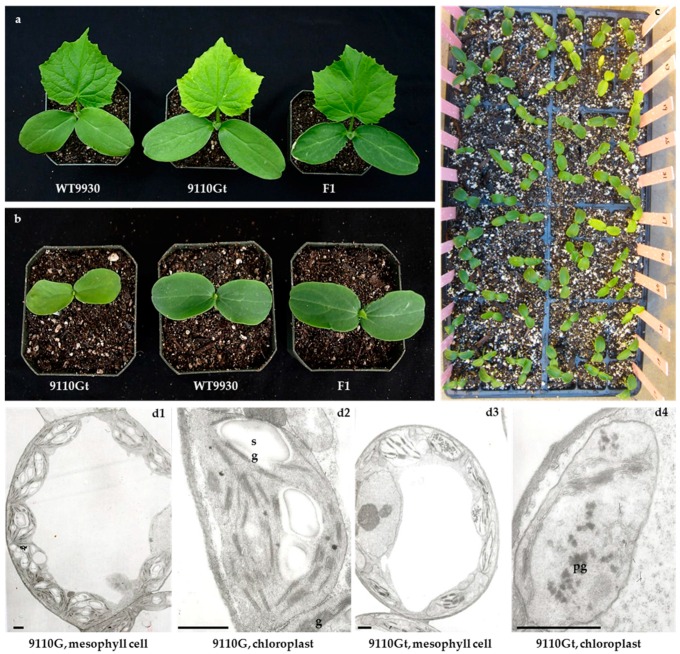
Phenotypic characterization of virescent leaf (*v-1*) mutant 9110Gt, wild type parental line 9930 and their F_1_. (**a**) Leaf color of 9110Gt, 9930, and their F_1_ at the first true leaf stage; (**b**) Cotyledon color of 9110Gt, 9930, and their F_1_; (**c**) Cotyledon colors of 9110Gt × 9930 F_2_ plants; (**d**) Structure of mesophyll cells and chloroplasts of 9110G and mutant 9110Gt. g = grana, sg = starch grains, pg = plastoglobulus, scale bar = 1 μm.

After germination, the virescent mutant 9110Gt showed light yellow cotyledons and first true leaf whereas those on the WT 9110G or 9930 were green (Figure 1a,b). As the true leaves became fully expanded, the light yellow color gradually turned to green. This trait was similar to the virescent leaf color (gene *v*) described by Pierce and Wehner [28]. The plant of 9110Gt was slightly smaller than that of WT 9110G throughout their development stages. We also found that this color change was temperature dependent. During the summer season, the yellow color in the seedlings was not as obvious as in the winter greenhouse season, and the duration of yellow color in the cotyledons and true leaves was shorter when the temperature was higher.

We previously showed that the virescent leaf phenotype of 9110Gt was controlled by a single recessive nuclear gene *v-1* [31]. In the present study, the F_1_ plants of both 9110Gt × 9110G and 9110Gt × 9930 had green leaves (Figure 1a,b); the 1800 F_2_ plants from the 9110Gt × 9930 cross segregated at 446 yellow leaf (*v-1v-1*) to 1354 green leaf (*V-1*_) (Figure 1c), which was consistent with the expected 3 green to 1 virescent segregating ratio in the *χ*^2^ test (*p* = 0.83) supporting a single recessive that was responsible for the virescent leaf color in 9110Gt.

We measured pigment (total chlorophyll and carotenoids) contents of 9110G and 9110Gt, and found that, at the virescent stage (cotyledon and first true leaf), the total chlorophyll (Chl) and carotenoids in 9110Gt mutant was only 44% and 21% of the wild-type 9110G, respectively (Table 1). This suggested that the *v-1* mutant phenotype is probably due to reduced contents of photosynthetic pigments. The decrease in Chla (46%) was greater than that for Chlb (35%) (Table 1), suggesting that the *v-1* mutant exhibits delayed greening during photomorphogenesis because of slower Chl accumulation. When the cotyledons and first true leaves were completely unfolded, mutant plants accumulated substantial quantities of chlorophyll, and the contents of Chl were not significantly different from those in the WT.

We examined the ultrastructure of chloroplasts in the leaves of 9110Gt and 9110G at the first true leaf stage, which are shown in Figure 1d. As compared with WT, 9110Gt exhibited fewer chloroplasts per cell and few thylakoids per chloroplast (Figure 1d). In addition, there were increased numbers of plastoglobuli, decreased numbers of starch granules, and a lack of granal membranes in the mesophyll chloroplasts of 9110Gt as compared with 9110G. These results indicated that the development of chloroplast was defective in the mutant.

### 2.2. High-Resolution Mapping of the v-1 Locus

**Figure 2:**
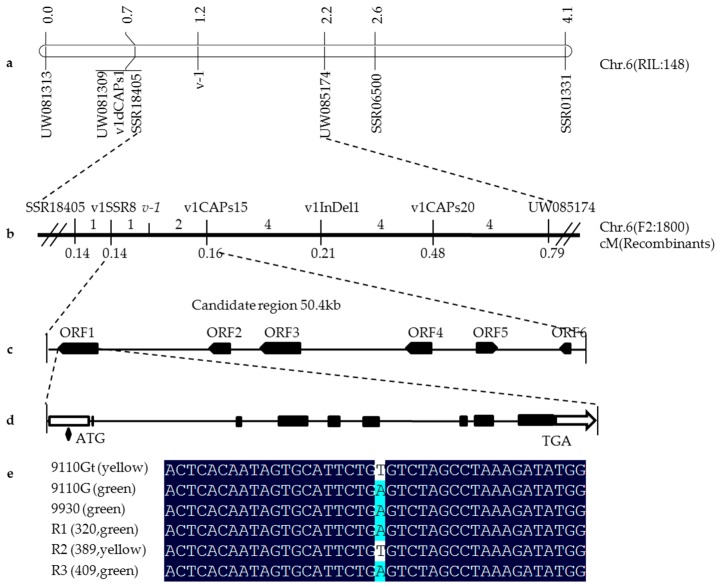
Fine genetic mapping of *v-1* gene. (**a**) Framework map of *v-1* gene with 148 RILs. Numbers above the chromosome are genetic distance in centiMorgan (cM); (**b**) Fine mapping narrowed the *v-1* locus into a 50.4 kb region flanked with markers v1SSR8 and CAPs15 based on 1800 F_2_ plants. Numbers above the chromosome are recombinants at each interval; (**c**) Six genes were predicted in the 50.4 kb region; (**d**) Structure of *CsaCNGCs* candidate gene. Black boxes are exons; white boxes are UTRs; and black lines indicate introns; and (**e**) Alleles of a SNP (A/T) in the promoter region of the *CsaCNGCs* candidate gene in 9110G, 9110Gt, 9930 and 3 recombinant plants.

Using 148 RILs of 9110Gt × 9930 cross, we previously mapped the *v-1* locus in cucumber chromosome 6 between SSR01331 and SSR18405 with genetic distances 1.6 and 0.3 cM, respectively [31]. Physically, both markers were located in the same scaffold from the 9930 cucumber draft genome assembly scaffold000066 and scaffold03904 in the Gy14 assembly, and the physical distance between the two markers was approximately 493 kb. To identify closer markers, based on the genomic DNA sequences of scaffold3904 and scaffold000066, 148SSR and five dCAPs markers were developed and screened for polymorphisms between 9930 and 9110Gt. Eleven polymorphic markers were identified and mapped with a polymorphism rate of 7.2%. Linkage analysis with 148 RILs suggested that UW085174 and SSR18405 were the two closet flanking markers, which were 1.2 and 0.3 cM away from the *v-1* locus, respectively (Figure 2a).

To fine map the *v-1* locus, the 1800 F_2_ plants were screened with UW081309, UW081313, UW085174, and SSR18405. The *v-1* gene was consistently mapped to an interval between UW085174 and SSR18405, and the order of these mapped loci was consistent with their physical positions in the scaffolds. The physical distance between UW085174 and SSR18405 was 171.5 kb in 9930 scaffold000066 and 171.9 kb in Gy14 scaffold03904. Of the 1800 F_2_ plants, 16 were recombinants between UW085174 and SSR18405. Examination of DNA sequence variations in this 172 kb region identified SNPs or indels that allowed design of 10 SSR, 1 indel, and 25 SNP-derived dCAPA/CAPS, and eventually 4 markers, v1SSR8,v1CAPs15, v1CAPs20, and v1InDel1 were mapped with the 16 F_2_ recombinant plants (Figure 2b). The names and sequence of all mapped markers on the map are listed in Supplemental Table S1. Now the *v-1* gene was delimited by markers v1SSR8 and v1CAPs15 with the genetic distance being 0.14 and 0.16 cM, respectively from *v-1*. The physical distance was approximately 50.4 kb in 9930 scaffold000066.

### 2.3. Candidate Gene Annotation and Sequence Analysis

Using the FGENESH program, we annotated the 50.4 kb genomic DNA sequences from 9930 scaffold000066, and six genes (ORF1 to ORF6) were predicted, which are listed in Table 2. None of the six genes have been reported to be involved in leaf color mutations in the literature. Annotation of ORF1 through ORF6 indicated that there were 8, 4, 9, 1, 1, and 2 exons in the coding regions of the six genes, respectively. Alignment of the CDS sequences of the six predicted genes revealed no nucleotide variations in ORF2 to ORF6 between 9110Gt and 9110G, suggesting that these five annotated genes are unlikely to be associated with the leaf color mutation. ORF1 was predicted to encode a cyclic nucleotide-gated channel (CNGC) protein (Table 2) which seemed to be a potential candidate gene for the *v-1* locus. Therefore, we designated the gene *CsaCNGCs*.

BLAST search of the cucumber Gy14 and 9930 draft genomes revealed a single copy of *CsaCNGCs* in the cucumber genome. We cloned the *CsaCNGCs* gene sequences from 9110Gt, 9930, and 9110G (Figure S1), which were 5010 bp in length. The coding region of *CsaCNGCs* gene was predicted to have eight exons and encode a protein with 720-amino acids (Figure 2e). Alignment of the *CsaCNGCs* candidate gene sequence identified no variations in the coding regions among 9110Gt, 9110G, and 9930 (Figure S1), but there was a single nucleotide mutation (A→T) in the promoter region before the transcription start site (TSS) (Figure 2e). There were three recombinants between the two markers v1SSR8 and v1CAPs15 flanking the *v-1* locus (Figure 2b). We genotyped the three recombinants by Sanger sequencing of the promoter region harboring this SNP and the result (Figure 2e) indicated that the SNP alleles were completely consistent with the leaf color phenotype, providing further evidence that *CsaCNGCs* was indeed the candidate gene for the *v-1* locus.

### 2.4. The Single Nucleotide Mutation in the Promoter Region at the CsaCNGCs Locus Was Unique in Different Cucumber Lines

**Figure 3:**
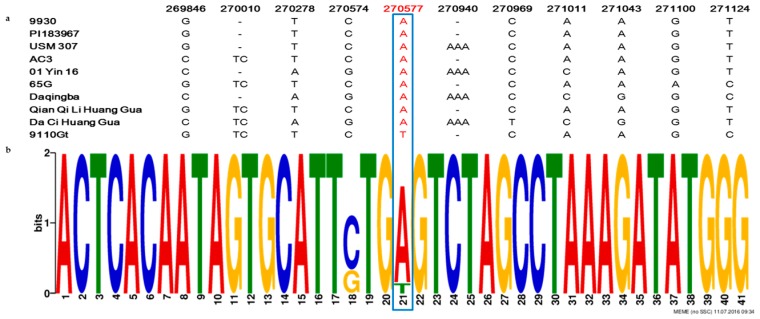
DNA sequence alignment of *CsaCNGCs* gene promoter regions among eight cucumber ecotypes. (PI 193967, USM 307, AC3, 01Yin16, 65G, Daqingba, Qian Qi Li Huang Gua, and Da Ci Huang Gua). (**a**) Among all SNPs or InDels detected in this region, only A→T SNP is consistent with the phenotypes (virescent vs. green leaf). The numbers on top are nucleotide positions in 9930 scaffold000066; (**b**) The single nucleotide mutation in the promoter region at the *CsaCNGCs* locus was unique in different cucumber lines. The numbers of sequences which −20 to +20 sequences of the single nucleotide mutation (A/T). Blue boxes = Unique single nucleotide mutation (A/T) in the promoter region of the *CsaCNGCs* candidate gene in 9110Gt.

To confirm the identity of the single nucleotide mutation in the promoter region at the *CsaCNGCs*, we investigated the sequence conservation at *CsaCNGCs* locus in eight cucumber lines of different ecotypes including a wild cucumber (*C. sativus* var.hardwickii, PI 193967), a south China type cucumber (USM 307), a North American pickling cucumber (AC3), two European greenhouse cucumbers (01Yin16 and 65G), and three north China type cucumbers (Daqingba, Qian Qi Li Huang Gua, and Da Ci Huang Gua). Motif searches in the 2000 bp sequence upstream of the *CsaCNGCs* TSS region in the eight cucumber lines revealed, nine SNPs, and two insertion-deletions nucleotide mutations in the promoter region (Figure 3a), butonly 9110Gt had a SNP (A→T) that was consistent with the virescent leaf phenotype among these lines (Figure 3b), supporting *CsaCNGCs* as the most possible candidate gene for the *v-1* locus.

### 2.5. Expression of CsaCNGCs in 9110Gt Mutant and 9110G WT

**Figure 4:**
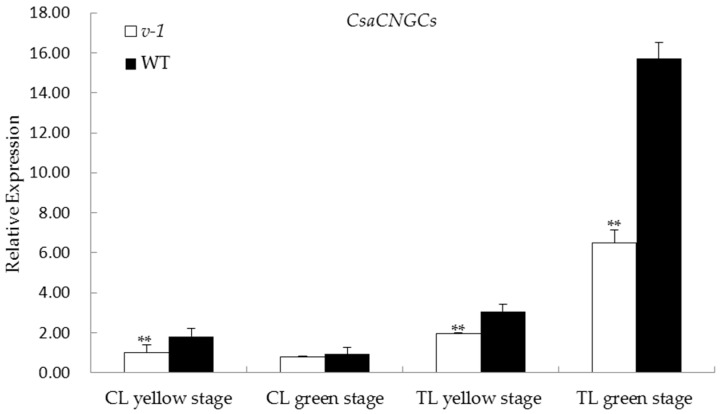
Real-time PCR expression analysis of *CsaCNGCs* candidate genes in 9110Gt (*v-1*) and 9110G (WT) at yellow and green stages. The relative expression level of each candidate gene was measured in the two lines at the cotyledon (CL) and the first true leaf (TL) stages when they were yellow and green in color by qPCR. EF1a was used as the internal control. Data are means ± SD (*n* = 3). Asterisks indicate statistically significant differences compared with the wild type at ** *p* < 0.05 by Student’s *t* test.

We quantified transcript abundance of *CsaCNGCs* in the cotyledons and first true leaves of 9110Gt and 9110G with qPCR at both virescent and green leaf stages. The forward and reverse primer sequences (5′-3′) for *CsaCNGCs* were: GGATTCATGGTTTAATACGAGC and GATTTTGTCCCAAGGAACTGA, respectively. The cucumber *EF1α* gene was used as the reference (left primer sequence: ACTGTGCTGTCCTCATTATTG; right: AGGGTGAAAGCAAGAAGAGC). The qPCR results are shown in Figure 4. It seemed that *CsaCNGCs* was constitutively expressed in cotyledons and the first true leaf. No difference in *CsaCNGCs* expression was found in the green cotyledons between WT and 9110Gt, but its expression was significantly lower in 9110Gt than in WT at both virescent and green first true leaves, as well as the virescent cotyledons. These observations may suggest that the virescent leaf color in 9110Gt is the result of reduced expression of *CsaCNGC*.

## 3. Discussion

The leaf-color mutation is a good marker for screening the genetic purity of hybrid crops [13]. It has been helpful understanding of chlorophyll anabolism and catabolism, chloroplast development, and photosynthesis [18]. A chloroplast is one of three types of plastids, characterized by its high concentration of chlorophyll. Plants with normal leaf colors have higher chlorophyll content and normal chloroplasts. In the present study, electron microscopic examination revealed delayed formation of thylakoid membranes in the chloroplasts of the 9110Gt virescent mutant (Figure 1d). The levels of chlorophyll a, chlorophyll b, and carotenoids in the cotyledons and first true leaves of 9110Gt at the virescent yellow stage were significantly lower than that in the wild type (9110G and 9930). Chlorophyll a was reduced more than chlorophyll b (Table 1). The malfunctioning chloroplast and reduced chlorophyll biosynthesis in yellow young leaves seem to be a common feature for many temperature-dependent virescent mutants such as those found in Arabidopsis [20], cotton [32]; rice [7,18,26,33,34,35,36,37,38], maize [39], wheat [40], and apple [41]. Biswal et al. [42] showed that the biosynthesis of chlorophyll a is closely linked to chloroplast development. Therefore, chlorophyll deficiency in the cotyledons and first leaves of 9110Gt may be attributed to delayed chloroplast development due to abnormal formation of thylakoid membranes.

In this study, mutant plant 9110Gt carrying the *v-1* gene have virescent leaves that exhibit yellowish-green leaves at the seedling stage, and then the color turns to normal green. Through map-based cloning, the *v-1* gene was finally delimited in a 50.4 kb region on the short arm of chromosome 6. On the same chromosome, only one gene *yp* [30] related to the leaf-color mutation has been mapped up to now. C528 mutants carrying the *yp* gene exhibited golden leaf color throughout their whole growth and development stage. According to the previous reports, this gene does not locate in the fine mapping region of *v-1*, so we think that these two mutants are controlled by different genes.

The promoter is the non-coding DNA region that occurs upstream of the coding region of a gene and is required for transcription of that specific gene. The promoter plays an important role in the process of plant gene expression and regulation [43]. Analysis of the *CsaCNGCs* gene sequence identified no variations in the coding regions among mutant and wildtype. We found a single nucleotide mutation from A to T in the promoter region of this candidate gene was the causal SNP for the viresent leaf mutation. In cucumber, the other only leaf color mutation cloned is the “golden leaf” controlled by the *yp* locus, which exhibited yellow color throughout its development stage [30]. Therefore, the present study represented the first report on map-based locating of a virescent leaf mutation in cucumber. Many genes that are responsible for virescent leaf mutations in different plant species have been cloned. Most of these genes function in chlorophyll biosynthesis and catabolism, or in the developmental regulation of chloroplast, in the metabolism of carotenoids [26,33,34,35,36]. In Arabidopsis [44], rice [36], and maize [39], several virescent mutants were due to nucleotide changes in the chloroplast Clp protease complex which play important roles in plastid development.

In the present study, from multiple lines of evidence, we showed that *CsaCNGCs*, a gene for the cyclic nucleotide-gated channel, were the only candidate gene for the *v-1* locus (Figure 2 and Figure 3). Cyclic-nucleotide-gated ion channels (CNGCs) are nonselective cation channels that were first identified in vertebrate retinal photoreceptors and olfactory sensory neurons [45]. In plants, it was first identified in a screen for calmodulin (CaM) binding partners in barley [46]. Arabidopsis has 20 CNGCs homologs [47], and their functions critically depend on the signaling molecules cAMP and/or cGMP [48]. Both cGMP and cAMP are reported to be involved in a number of physiological processes such as chloroplast development, stomatal functioning, monovalent and divalent cation fluxes, gibberellic acid signaling, pathogen response, and gene transcription [49,50]. The cyclic GMP is able to trigger the production of anthocyanins, and that a combination of cyclic GMP with calcium can regulate the development of chloroplasts and the photosynthetic machinery [51]. *CsaCNGCs* shared 64% amino acid sequence identity with Arabidopsis *AtCNGC15*, and the expression level of *CsaCNGCs* in WT plants was higher than in the mutant (Figure 4).

The mechanism underlying the leaf color change from yellow to green in virescent leaf mutants is an interesting question. In the rice virescent mutant v14, the second and third leaves displayed an albino phenotype, but from the fourth leaf stage, all leaves including the albino ones are recovered to normal green [38]. The cells of the second and third leaves of the v14 mutant lack mature thylakoid membrane systems and starch grain but normal chloroplast structure can be observed in the fourth leaf of the same mutant. Zhang et al. [38] reasoned that there may be different regulation pathways that are involved in chloroplast development in rice. Another possible explanation is that there might be other *CsaCNGCs* homologs with redundant functional activities in greening stages. The connection between reduced *CsaCNGCs* expression with chlorophyll deficiency, delayed chloroplast development, and the virescent leaf in the *v-1* mutant is not known and merits further investigation.

## 4. Materials and Methods

### 4.1. Plant Materials

Two cucumber inbred lines, 9110Gt and 9930, were used for phenotypic characterization of the virescent mutant and development of segregating populations. 9110Gt was a spontaneous virescent leaf mutation found in 9110G, which has a largely European greenhouse cucumber genetic background [29]. 9930 is a typical north China fresh market cucumber, and the draft genome of 9930 is available [52]. For genetic mapping, 148 recombinant inbred lines (RILs) and 1800 F_2_ individuals from the cross between 9110Gt and 9930 were developed. For phenotyping, all plant materials were grown in the greenhouses of the Institute of Vegetables and Flowers (IVF), Chinese Academy of Agricultural Sciences (Beijing, China) or the Walnut Street Greenhouse of the University of Wisconsin (Madison, WI, USA) in 2012 to 2014 greenhouse seasons. The temperature in the greenhouses was 25–30 °C during the day and 18–25 °C in the night with 30%–85% relative humidity. Leaf-color phenotypes in the RIL and F_2_ populations were scored before three-true-leaf seedling stage (before as either green (wild type) or virescent yellow (mutant) [31].

### 4.2. Measurement of Chlorophyll Contents

Fresh leaves (0.2 g) of 9110Gt mutant and 9100G wild type (WT) seedlings were soaked in 80% acetone and the absorption of the extract measured in a Shimadzu UV-1700 UV-visible spectrophotometer at 646, 663, and 470 nm wavelengths for chlorophyll a/b (Chla, Chlb) and carotenoids (Caro), respectively. The concentrations of total chlorophyll and carotenoids were calculated as described by Lichtenthaler [53].

### 4.3. Transmission Electron Microscopy

Fresh leaf sections were fixed in 3.0% glutaraldehyde solution followed by 1.0% Osmium tetroxide (OsO_4_). The tissues were dehydrated in a gradient acetone series and embedded in Epon618 epoxy resin prior to thin sectioning. After staining with uranyl acetate and Reynolds’ lead citrate, the sections were observed under a Hitachi H7500 transmission electron microscope (Tokyo, Japan).

### 4.4. Genomic DNA Extraction, PCR, and Electrophoresis

Genomic DNA was isolated from young leaf tissues with the CTAB method [31]. The DNA was dissolved in ddH_2_O and kept at −20 °C until use. A UV spectrophotometer (Shanghai, China) was used to measure DNA purity and concentration, and the final concentration of DNA was diluted to 30 ng/μL. The polymerase chain reaction (PCR) system and protocol was the same with Miao et al. [31]. PCR amplified products were separated on 6.0% non-denaturing polyacrylamide gels in 1× TBE buffer at 150 V for 1.5–2 h, and the bands were visualized and photographed after silver staining [54].

### 4.5. Development of Molecular Markers for Fine Mapping

In our previous study, with 148 RILs, we showed that the virescent leaf of 9110Gt was controlled by a single recessive nuclear gene *v-1*, which was place in cucumber chromosome 6 flanked with two microsatellite markers SSR01331 and SSR18405 that were 1.6 cM and 0.3 cM away from the *v-1* locus, respectively. A scaffold-based chromosome walking strategy was taken to identify more closely linked markers [55]. Draft genome scaffold assemblies from both the 9930 (V2.0) [52] and Gy14 (V1.0) [56] cucumber lines were employed. In the target scaffolds, new markers were selected from the published cucumber genetic maps [57,58] and a collection of 83,689 SSR markers that were developed from the Gy14 draft genome assembly [59].

At the fine mapping stage, SNPs and indels were explored in the next-generation whole genome re-sequencing data. The genomes of 9110Gt, Gy14, and 9930 cucumber inbred lines were re-sequenced with the Illumina Hi-Seq 2000 at >15× coverage each (100 bp paired end). For marker discovery, short sequencing reads were aligned to the 9930 draft genome with the BWA (Burrows-Wheeler Alignment Tool) software package [60]. Indel identification and SNP-calling were performed by SAM tools [61]. For SNP genotyping, dCAPs and CAPs markers were developed with dCAPS Finder 2.0 [62]. A total of 189 new SSR, dCAPs, CAPs, and InDel markers were developed. Primer design was performed with Primer 3.0 (http://bioinfo.ut.ee/primer3-0.4.0) [63]. All primers were synthesized commercially or at the Biotech Center at the University of Wisconsin at Madison. All newly developed markers were first screened for polymorphism with the two parental lines; polymorphic markers were applied to the recombinants in the RIL population for linkage analysis, which were then extended to the larger F_2_ population.

### 4.6. Linkage Mapping and Candidate Gene Annotation

Linkage analysis between DNA markers and the *v-l* locus was performed with JoinMap 4.0 with a LOD threshold of 3.0. The recombination percentages were converted to genetic distances using the Kosambi mapping function [64]. Annotation of DNA sequences in the target genomic DNA region was performed using the program FGENESH (http://linux1.softberry.com/berry.phtml) [65], and the results were checked manually. Gene prediction was conducted with BLASTx at the NCBI (National Center for Biotechnology Information) website (http://blast.ncbi.nlm.nih.gov). Re-sequencing reads of different cucumber lines were aligned with the BWA software using the 9930 draft genome as the reference. To detect potential promoter motifs, sequence regions corresponding to upstream of the TSS positions were scanned by MEME (Motif-based sequence analysis tools) version 4.11.2 (http://meme-suite.org/tools/meme).

### 4.7. Real-Time Quantitative PCR of Candidate Genes

The cotyledons and first true leaves of 9110Gt and 9110G were flash frozen in liquid nitrogen and used for total RNA extraction using TRIzol^®^ (Thermo Fisher Scientific, Waltham, MA, USA). RNA was pretreated with RNase-free DNase I (Takara, Shiga, Japan), and first-strand cDNA was synthesized from 1.0 μg total RNA using reverse transcriptase SYBR FAST qPCR Kit Master Mix (2×) Universal (KAPA Biosystems, Wilmington, MA, USA).

Quantitative real time PCR (qPCR) was performed in a total volume of 10.0 μL which contained 1.0 μL of first-strand cDNA, 5.0 μL SYBR FAST qPCR Kit Master Mix, 0.4 μL gene-specific primers (10 μM·μL^−1^), 0.2 μL ROX reference dye, and 3.4 μL ddH2O. Reactions were amplified in a ABI prism 7900 HT Real-Time PCR System with following conditions: pre-incubation at 95 °C for 3 min; 40 cycles of 95 °C for 30 s, 60 °C for 20 s; melting curves 95 °C for 15 s, 60 °C for 15 s, and 95 °C for 15 s. The 2^ΔΔ*C*t^ method [66] was used to calculate relative changes in gene expression. All runs had three technical replicates and three biological replicates.

## Figures and Tables

**Table 1:** Photosynthetic pigment content in virescent leaf mutant 9110Gt and 9110G (WT) at the yellowing stage (Chl = chlorophyll, Car = carotenoid; mg/g FW).

Materials	Chl*a* (mg/g)	Chl*b* (mg/g)	Caro (mg/g)	Total Chl (mg/g)	Chl*a/b*
9110G	1.38 ± 0.11 **	0.40 ± 0.01 **	0.39 ± 0.01 *	1.78 ± 0.01 **	3.45
9110Gt	0.74 ± 0.02	0.26 ± 0.01	0.31 ± 0.06	1.00 ± 0.21	2.85
% of WT	46.0	35.0	21.0	44.0	17.0

*, **: Significant at 5% and 1% level, respectively.

**Table 2:** Information of six predicted genes in the 50.4 kb region of the *v-1* locus.

Gene	Start	End	Exon	Annotation
ORF1	263878	268887	8	cyclic nucleotide-gated channel
ORF2	277911	280354	4	3′-5′-exoribonuclease family protein
ORF3	281291	286306	9	receptor-like serine/threonine-protein kinase NCRK
ORF4	289805	293123	1	uncharacterized protein
ORF5	293407	294673	1	uncharacterized protein
ORF6	299479	301054	2	expansin A23

## Supplementary Materials

Supplementary materials can be found at www.mdpi.com/1422-0067/17/10/1602/s1.
