# Supplementary material for: Fine Mapping of Virescent Leaf Gene v-1 in Cucumber (Cucumis sativus L.)
Source: Int J Mol Sci. 2016 Sep 22;17(10):1602. doi: 10.3390/ijms17101602 (PMC5085635; doi:10.3390/ijms17101602)
Supplement: Supplementary file 1 [file ijms-17-01602-s001.pdf]

Supplementary Materials: Fine Mapping of Virescent Leaf Gene *v-1* in Cucumber (*Cucumis sativus* L.)

Han Miao, Shengping Zhang, Min Wang, Ye Wang, Yiqun Weng and Xingfang Gu

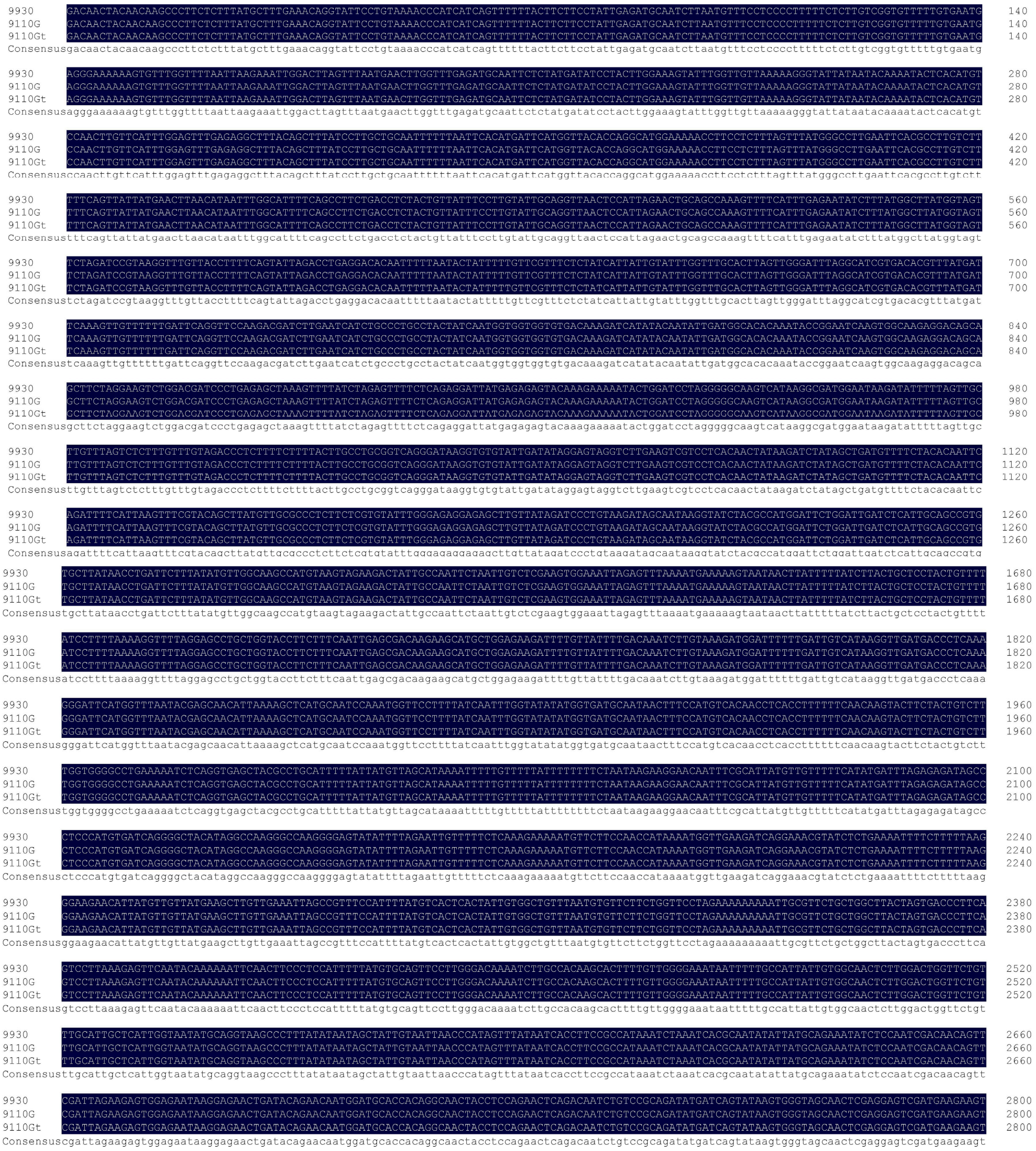

Figure S1. *Cont.*

```
9930     GCTGCTGAGAAGCCTTCCCTTGGATCTCCGGAGAGATATAAAACGTCACCTTTGCCTTGACCTCGTTCGAAGGGTAAATTTCACATATAAATTTGCATGACATATGGATACGGAGGAGCAGTGTTGGAAATGACTGCTCA  2940
9110G    GCTGCTGAGAAGCCTTCCCTTGGATCTCCGGAGAGATATAAAACGTCACCTTTGCCTTGACCTCGTTCGAAGGGTAAATTTCACATATAAATTTGCATGACATATGGATACGGAGGAGCAGTGTTGGAAATGACTGCTCA  2940
9110Gt   GCTGCTGAGAAGCCTTCCCTTGGATCTCCGGAGAGATATAAAACGTCACCTTTGCCTTGACCTCGTTCGAAGGGTAAATTTCACATATAAATTTGCATGACATATGGATACGGAGGAGCAGTGTTGGAAATGACTGCTCA  2940
Consensusgctgctgagaagccttcccttggatctccggagagatataaaacgtcacctttgccttgacctcgttcgaagggtaaatttcacatataaatttgcatgacatatggatacggaggagcagtgttggaaatgactgctca

9930     CGAATTACAATTTGTCGCAAATTAAACCGATTCATCTCTGTGCACCCCCTTCTTGATCTTTGCAGGTTCCATTGTTTGATCAAATGGATGAAAGAATGCTAGATGCAATATGTGAGAGACTTAAACCCGCTTTGAGCACA  3080
9110G    CGAATTACAATTTGTCGCAAATTAAACCGATTCATCTCTGTGCACCCCCTTCTTGATCTTTGCAGGTTCCATTGTTTGATCAAATGGATGAAAGAATGCTAGATGCAATATGTGAGAGACTTAAACCCGCTTTGAGCACA  3080
9110Gt   CGAATTACAATTTGTCGCAAATTAAACCGATTCATCTCTGTGCACCCCCTTCTTGATCTTTGCAGGTTCCATTGTTTGATCAAATGGATGAAAGAATGCTAGATGCAATATGTGAGAGACTTAAACCCGCTTTGAGCACA  3080
Consensuscgaattacaatttgtcgcaaattaaaccgattcatctctgtgcacccccttcttgatctttgcaggttccattgtttgatcaaatggatgaaagaatgctagatgcaatatgtgagagacttaaacccgctttgagcaca

9930     GAAGGCACATTTCTTGTGCGAGAAGGTGACCCCGTCAATGAAATGCTATTCATAATCAGAGGTCATCTTGACTCATACACCACTAACGGTGGTAGAACTGGGTTTTTCAACTCCTGTCGTATTGGTCCGGGTGATTTCTG  3220
9110G    GAAGGCACATTTCTTGTGCGAGAAGGTGACCCCGTCAATGAAATGCTATTCATAATCAGAGGTCATCTTGACTCATACACCACTAACGGTGGTAGAACTGGGTTTTTCAACTCCTGTCGTATTGGTCCGGGTGATTTCTG  3220
9110Gt   GAAGGCACATTTCTTGTGCGAGAAGGTGACCCCGTCAATGAAATGCTATTCATAATCAGAGGTCATCTTGACTCATACACCACTAACGGTGGTAGAACTGGGTTTTTCAACTCCTGTCGTATTGGTCCGGGTGATTTCTG  3220
Consensusgaaggcacatttcttgtgcgagaaggtgaccccgtcaatgaaatgctattcataatcagaggtcatcttgactcatacaccactaacggtggtagaactgggtttttcaactcctgtcgtattggtccgggtgatttctg

9930     TGGTGAGGAGCTGCTGACTTGGGCTCTTGACCCTCGTCCAAGCGTTGTGCTTCCGTCTTCAACTCGCACGGTCAAAGCCATCTCCGAGGTAGAAGCATTTGCTCTCATAGCCGAGGACTTGAAATTTGTATCCTCCCAGT  3360
9110G    TGGTGAGGAGCTGCTGACTTGGGCTCTTGACCCTCGTCCAAGCGTTGTGCTTCCGTCTTCAACTCGCACGGTCAAAGCCATCTCCGAGGTAGAAGCATTTGCTCTCATAGCCGAGGACTTGAAATTTGTATCCTCCCAGT  3360
9110Gt   TGGTGAGGAGCTGCTGACTTGGGCTCTTGACCCTCGTCCAAGCGTTGTGCTTCCGTCTTCAACTCGCACGGTCAAAGCCATCTCCGAGGTAGAAGCATTTGCTCTCATAGCCGAGGACTTGAAATTTGTATCCTCCCAGT  3360
Consensustggtgaggagctgctgacttgggctcttgaccctcgtccaagcgttgtgcttccgtcttcaactcgcacggtcaaagccatctccgaggtagaagcatttgctctcatagccgaggacttgaaatttgtatcctcccagt

9930     TCAGAAGACTCCACAGCAAACAACTAAGACACAAGTTTCGATTCTACTCGCATCAATGGCGAACATGGGCTGCATGCTTTGTTCAAGCAGCATGGCGAAGATATAAGAGACGGAAAGAGCGAGCTGAACTCAGGGCTAAA  3500
9110G    TCAGAAGACTCCACAGCAAACAACTAAGACACAAGTTTCGATTCTACTCGCATCAATGGCGAACATGGGCTGCATGCTTTGTTCAAGCAGCATGGCGAAGATATAAGAGACGGAAAGAGCGAGCTGAACTCAGGGCTAAA  3500
9110Gt   TCAGAAGACTCCACAGCAAACAACTAAGACACAAGTTTCGATTCTACTCGCATCAATGGCGAACATGGGCTGCATGCTTTGTTCAAGCAGCATGGCGAAGATATAAGAGACGGAAAGAGCGAGCTGAACTCAGGGCTAAA  3500
Consensustcagaagactccacagcaaacaactaagacacaagtttcgattctactcgcatcaatggcgaacatgggctgcatgctttgttcaagcagcatggcgaagatataagagacggaaagagcgagctgaactcagggctaaa

9930     GAGAGTTATCCAACTACCAAAGTCGAGTCCCCGCTTTCACCACCAACGACATCAAACTTGGATTCACGATCAGGAAATGCTAGAAGGGCATTGAATAAGCGTTGTGTTTCAGATGCTGGGGTCGTAAGCTCATTGCAGAA  3640
9110G    GAGAGTTATCCAACTACCAAAGTCGAGTCCCCGCTTTCACCACCAACGACATCAAACTTGGATTCACGATCAGGAAATGCTAGAAGGGCATTGAATAAGCGTTGTGTTTCAGATGCTGGGGTCGTAAGCTCATTGCAGAA  3640
9110Gt   GAGAGTTATCCAACTACCAAAGTCGAGTCCCCGCTTTCACCACCAACGACATCAAACTTGGATTCACGATCAGGAAATGCTAGAAGGGCATTGAATAAGCGTTGTGTTTCAGATGCTGGGGTCGTAAGCTCATTGCAGAA  3640
Consensusgagagttatccaactaccaaagtcgagtccccgctttcaccaccaacgacatcaaacttggattcacgatcaggaaatgctagaagggcattgaataagcgttgtgtttcagatgctggggtcgtaagctcattgcagaa

9930     GCCAGAAGAACCTGACTTCTCCATTGTTGAGGAGTAAAGTGACGATGGAGCTCGGCTGAAAAACCAGCAACTGAGTCTGTACAGGTGGGGTGTGAACGGTGAAGCTATACTTACTACTGGTTGTTTTGTGTAAAATGAAA  3780
9110G    GCCAGAAGAACCTGACTTCTCCATTGTTGAGGAGTAAAGTGACGATGGAGCTCGGCTGAAAAACCAGCAACTGAGTCTGTACAGGTGGGGTGTGAACGGTGAAGCTATACTTACTACTGGTTGTTTTGTGTAAAATGAAA  3780
9110Gt   GCCAGAAGAACCTGACTTCTCCATTGTTGAGGAGTAAAGTGACGATGGAGCTCGGCTGAAAAACCAGCAACTGAGTCTGTACAGGTGGGGTGTGAACGGTGAAGCTATACTTACTACTGGTTGTTTTGTGTAAAATGAAA  3780
Consensusgccagaagaacctgacttctccattgttgaggagtaaagtgacgatggagctcggctgaaaaaccagcaactgagtctgtacaggtggggtgtgaacggtgaagctatacttactactggttgttttgtgtaaaatgaaa

9930     GTTTGGTTAAGAAAAATTTACGTGGGATTTTC  3813
9110G    GTTTGGTTAAGAAAAATTTACGTGGGATTTTC  3813
9110Gt   GTTTGGTTAAGAAAAATTTACGTGGGATTTTC  3813
Consensusgtttggttaagaaaaatttacgtgggattttc
```

Figure S1. *CsaCNGs* DNA sequence from 3 cucumbers as follow. 9930, 9110G and 9110Gt.

Table S1. Information of molecular makers used in fine mapping of *v*-1 in this study.

#	Marker	Marker Types	9930 Scaffold	Position	Chr.	Forward Primer (5′-3′)	Reverse Primer (5′-3′)	Enzyme
1	UW081313	SSR	scaffold000066	166409	6	CACGTGTGTTTCACAACTTTCTT	TTATCGACCAATTGCCAACA	
2	UW081309	SSR	scaffold000066	170584	6	TTTGGAAATAGCAACCTATGAGC	GAACCCAACTAATGCCAAAGA	
3	v1dCAPs1	dCAPs	scaffold000066	194393	6	TGCAAAGTTTGTTGCATTTGA	GGGGCATTAAGTTTGCTTGA	HaeIII
4	SSR18405	SSR	scaffold000066	206319	6	CGCAGGTGCATCTCATGTAA	GACAAACAAGGGGACGAAAA	
5	v1SSR8	SSR	scaffold000066	249054	6	GGCAATGGTAAAGGTTGGAA	AAGGGATGTGTGGTGTGGTT	
6	v1CAPs15	CAPS	scaffold000066	299463	6	ATCTCACTTTCATCTATTTATTGAC	TTGGCCAGAACTTTGTAGCA	MaeII
7	v1InDel1	InDel	scaffold000066	330800	6	GAGATACATGCAAAATTAGCAA	TGTGATGTCCATGATGAGATTG	
8	v1CAPs20	CAPS	scaffold000066	340002	6	GTATAATGAGAAAATAAATATGA	CCAATTCAAACATGCTCATTG	SspI
9	v1CAPs23	CAPS	scaffold000066	374798	6	AGTTCGCCTTAATCAACTCACTAAA	GGCCTCTACTCACCCTGAAA	HindIII
10	UW085174	SSR	scaffold000066	377830	6	TCCAAAACAAGAGACGACCA	AAGGGTGGGAGAAAAATGAA	
11	SSR06500	SSR	scaffold000066	414339	6	TGACAAAACACTACCCACCG	AGCAAATTCAACCGTTCATGT	
12	SSR01331	SSR	scaffold000066	699190	6	CGGGATTTACCCCTCACATT	GTGGGACCGAGAAGTTTGAT	
